# Supplementary material for: Classification of large ornithopod dinosaur footprints using Xception transfer learning
Source: PLoS One. 2023 Nov 29;18(11):e0293020. doi: 10.1371/journal.pone.0293020 (PMC10686485; doi:10.1371/journal.pone.0293020)

PLOS ONE

Classification of large ornithopod dinosaur footprints using Xception transfer learning

--Manuscript Draft--

**Manuscript Number:**	PONE-D-23-08114
**Article Type:**	Research Article
**Full Title:**	Classification of large ornithopod dinosaur footprints using Xception transfer learning
**Short Title:**	Machine learning classification of large ornithopod dinosaur footprints
**Corresponding Author:**	Seung-Sep Kim<br>Chungnam National University<br>Daejeon, KOREA, REPUBLIC OF
**Keywords:**	machine learning; classification; ornithopod; footprints
**Abstract:**	Large ornithopod dinosaur footprints have been confirmed on all continents except Antarctica since the 19th century. However, oversplitting problems in ichnotaxa have historically been observed in these footprints. To address these issues and distinguish between validated ichnotaxa, this study employed convolutional neural network-based Xception transfer learning to automatically classify ornithopod dinosaur tracks. The machine learning model was trained for 162 epochs using 274 data images, excluding horizontally flipped images. The trained model accuracy was 96.36%, and the validation accuracy was 92.59%. We demonstrate the performance of the machine learning model using footprint illustrations that are not included in the training dataset. These results show that the machine learning model developed in this study can properly classify footprint illustration data for large ornithopod dinosaurs. However, the quality of footprint illustration data (or images) inherently affects the performance of our machine learning model, which performs better on well-preserved footprints. In addition, because the developed machine-learning model is a typical supervised learning model, it is not possible to introduce a new label or class. Although this study used illustrations rather than photos or 3D data, it is the first application of machine-learning techniques at the academic level for large ornithopod dinosaur footprints. Furthermore, the machine learning model will likely prevent the misnaming of the large ornithopod dinosaur footprint ichnotaxa, thereby safeguarding against the oversplitting problem.
**Order of Authors:**	Yeoncheol Ha
	Seung-Sep Kim
**Additional Information:**	
**Question**	**Response**
**Financial Disclosure**<br><br>Enter a financial disclosure statement that describes the sources of funding for the work included in this submission. Review the submission guidelines for detailed requirements. View published research articles from *PLOS ONE* for specific examples.<br><br>This statement is required for submission and **will appear in the published article** if the submission is accepted. Please make sure it is accurate.	This study was supported by the National Research Foundation of Korea (NRF-2021R1A4A5026233 and NRF-2021R1A2C1012030), awarded to S.-S.K. The funder had no role in study design, data collection and analysis, decision to publish, or preparation of the manuscript.

Powered by Editorial Manager® and ProduXion Manager® from Aries Systems Corporation

**Unfunded studies**<br>Enter: *The author(s) received no specific funding for this work.*<br><br>**Funded studies**<br>Enter a statement with the following details:<br>• Initials of the authors who received each award<br>• Grant numbers awarded to each author<br>• The full name of each funder<br>• URL of each funder website<br>• Did the sponsors or funders play any role in the study design, data collection and analysis, decision to publish, or preparation of the manuscript?<br>• **NO** - Include this sentence at the end of your statement: *The funders had no role in study design, data collection and analysis, decision to publish, or preparation of the manuscript.*<br>• **YES** - Specify the role(s) played.<br><br>* typeset	
**Competing Interests**<br><br>Use the instructions below to enter a competing interest statement for this submission. On behalf of all authors, disclose any competing interests that could be perceived to bias this work—acknowledging all financial support and any other relevant financial or non-financial competing interests.<br><br>This statement is required for submission and **will appear in the published article** if the submission is accepted. Please make sure it is accurate and that any funding sources listed in your Funding Information later in the submission form are also declared in your Financial Disclosure statement.<br><br>View published research articles from *PLOS ONE* for specific examples.	The authors have declared that no competing interests exist.

Powered by Editorial Manager® and ProduXion Manager® from Aries Systems Corporation

**NO authors have competing interests**<br><br>Enter: *The authors have declared that no competing interests exist.*<br><br>**Authors with competing interests**<br><br>Enter competing interest details beginning with this statement:<br><br>*I have read the journal's policy and the authors of this manuscript have the following competing interests: [insert competing interests here]*<br><br>* typeset	
**Ethics Statement**<br><br>Enter an ethics statement for this submission. This statement is required if the study involved:<br><br>• Human participants<br>• Human specimens or tissue<br>• Vertebrate animals or cephalopods<br>• Vertebrate embryos or tissues<br>• Field research<br><br>Write "N/A" if the submission does not require an ethics statement.<br><br>General guidance is provided below. Consult the submission guidelines for detailed instructions. **Make sure that all information entered here is included in the Methods section of the manuscript.**	N/A

**Format for specific study types**<br><br>**Human Subject Research (involving human participants and/or tissue)**<br>• Give the name of the institutional review board or ethics committee that approved the study<br>• Include the approval number and/or a statement indicating approval of this research<br>• Indicate the form of consent obtained (written/oral) or the reason that consent was not obtained (e.g. the data were analyzed anonymously)<br><br>**Animal Research (involving vertebrate animals, embryos or tissues)**<br>• Provide the name of the Institutional Animal Care and Use Committee (IACUC) or other relevant ethics board that reviewed the study protocol, and indicate whether they approved this research or granted a formal waiver of ethical approval<br>• Include an approval number if one was obtained<br>• If the study involved *non-human primates*, add *additional details* about animal welfare and steps taken to ameliorate suffering<br>• If anesthesia, euthanasia, or any kind of animal sacrifice is part of the study, include briefly which substances and/or methods were applied<br><br>**Field Research**<br><br>Include the following details if this study involves the collection of plant, animal, or other materials from a natural setting:<br>• Field permit number<br>• Name of the institution or relevant body that granted permission	
**Data Availability**<br><br>Authors are required to make all data underlying the findings described fully available, without restriction, and from the time of publication. PLOS allows rare exceptions to address legal and ethical concerns. See the PLOS Data Policy and FAQ for detailed information.	Yes - all data are fully available without restriction

Powered by Editorial Manager® and ProduXion Manager® from Aries Systems Corporation

A Data Availability Statement describing where the data can be found is required at submission. Your answers to this question constitute the Data Availability Statement and **will be published in the article**, if accepted.<br><br>**Important:** Stating 'data available on request from the author' is not sufficient. If your data are only available upon request, select 'No' for the first question and explain your exceptional situation in the text box.<br><br>Do the authors confirm that all data underlying the findings described in their manuscript are fully available without restriction?	
**Describe where the data may be found in full sentences. If you are copying our sample text, replace any instances of XXX with the appropriate details.**<br><br>• If the data are **held or will be held in a public repository**, include URLs, accession numbers or DOIs. If this information will only be available after acceptance, indicate this by ticking the box below. For example: *All XXX files are available from the XXX database (accession number(s) XXX, XXX.*).<br>• If the data are all contained **within the manuscript and/or Supporting Information files**, enter the following: *All relevant data are within the manuscript and its Supporting Information files.*<br>• If neither of these applies but you are able to provide **details of access elsewhere**, with or without limitations, please do so. For example:<br><br>*Data cannot be shared publicly because of [XXX]. Data are available from the XXX Institutional Data Access / Ethics Committee (contact via XXX) for researchers who meet the criteria for access to confidential data.*<br><br>*The data underlying the results presented in the study are available from (include the name of the third party*	All relevant data are within the manuscript.

and contact information or URL).<br>• This text is appropriate if the data are owned by a third party and authors do not have permission to share the data.<br><br>* typeset	
Additional data availability information:	Tick here if the URLs/accession numbers/DOIs will be available only after acceptance of the manuscript for publication so that we can ensure their inclusion before publication.

Powered by Editorial Manager® and ProduXion Manager® from Aries Systems Corporation

Manuscript
Click here to access/download;Manuscript;YCHa_paper_230317_final_Ha_ss

Classification of large ornithopod dinosaur footprints using Xception transfer learning

Yeoncheol Ha[1], Seung-Sep Kim[1,2,*]

[1]Department of Astronomy, Space Science and Geology, Chungnam National University, Daejeon 34134, Korea

[2]Department of Geological Sciences, Chungnam National University, Daejeon 34134, Korea

* Corresponding author

E-mail: seungsep@cnu.ac.kr

Abstract

Large ornithopod dinosaur footprints have been confirmed on all continents except Antarctica since the 19th century. However, oversplitting problems in ichnotaxa have historically been observed in these footprints. To address these issues and distinguish between validated ichnotaxa, this study employed convolutional neural network-based Xception transfer learning to automatically classify ornithopod dinosaur tracks. The machine learning model was trained for 162 epochs using 274 data images, excluding horizontally flipped images. The trained model accuracy was 96.36%, and the validation accuracy was 92.59%. We demonstrate the performance of the machine learning model using footprint illustrations that are not included in the training dataset. These results show that the machine learning model developed in this study can properly classify footprint illustration data for large ornithopod dinosaurs. However, the quality of footprint illustration data (or images) inherently affects the performance of our machine learning model, which performs better on well-preserved footprints. In addition, because the developed machine-learning model is a typical supervised learning model, it is not possible to introduce a new label or class. Although this study used illustrations rather than photos or 3D data, it is the first application of machine-learning techniques at the academic level for large ornithopod dinosaur footprints. Furthermore, the machine learning model will likely prevent the misnaming of the large ornithopod dinosaur footprint ichnotaxa, thereby safeguarding against the oversplitting problem.

Introduction

Dinosaur footprints provide paleoecological information about pace, stride, speed, gait, and behavioral characteristics that body fossils cannot provide [1]. Historically, large ornithopod dinosaur footprints were associated with *Iguanodon* or *Iguanodontidae* [2], and have been confirmed from the 19th century to the present on all continents except Antarctica [3]. However, oversplitting issues (where species or other taxonomic groups are divided into too many smaller groups based on minor differences) of ichnotaxa in large ornithopod dinosaur footprints have been recognized [2–5] due to various factors. For instance, the definition of ichnotaxa is sometimes based on poorly preserved samples or lacks diagnostic features [6], inadequate diagnostics [2], or temporal or geographic criteria [3,7]. In addition, marking and confirming the outline of dinosaur footprints with a material such as chalk [8–11] can be affected by the researchers' subjectivity and experience, as well as the outlining method.

To circumvent such issues, 3D techniques such as photogrammetry and light detection and ranging (LiDAR) have recently been introduced for digitizing and recording dinosaur footprints [10–26]. Furthermore, Lallensack [27] produced a program to form continuous dinosaur footprints in R environment. Although these studies can effectively alleviate the significance of subjectively recording and digitizing dinosaur footprints, the oversplitting problem associated with the classification of large ornithopod ichnotaxa has not yet been fully addressed.

For example, the scientific names of the large ornithopod dinosaur ichnotaxa have been reviewed and renamed. Based on their overall morphology [2], large ornithopod footprints have been classified as *Amblydactylus gethingi* [28], *Caririchnium* [29], *Iguanodontipus* [6], and *Hadrosauropodus* [2]. The Early Cretaceous large ornithopod dinosaur footprints were classified

as *Iguanodontipus* (footprints of *Iguanodon*), *Amblydactylus* (strictly bipedal), and *Caririchnium* (quadrupedal) [30]. However, one study argued that all large ornithopod dinosaur footprints should be classified as *Amblydactylus* (*Iguanodontipus*) or *Caririchnium* (=*Hadrosauropodus*) [5], although the study failed to differentiate between *Hadrosauropodus* [2] and *Caririchnium*. Another study classified ornithopod dinosaur footprints into *Iguanodontipus*, *Caririchnium*, and *Hadrosauropodus* based on their biochronological and morphological parameters, including footprint size [4]. However, a recent study defined only *Iguanodontipus*, *Caririchnium*, and *Hadrosauropodus* among 34 ornithopod dinosaur footprints as valid ornithopod ichnotaxa, based on the size of the pes, heel, and digit impressions, which exhibit mesaxony, tridactyl, and subsymmetrical characteristics [3]. Fig 1 summarizes the changes in classification schemes over the last two decades. In this study, we employed the classification criteria of Díaz-Martínez et al. [3] to develop a machine learning classification method for large ornithopod dinosaur footprints (Table 1).

Table 1. Large ornithopod footprint classification validated by Díaz-Martínez et al. [3].

Previous classification	Díaz-Martínez et al. [3]
Amblydactylus kortmeyeri	*Caririchnium kortmeyeri*
Caririchnium magnificum	*Caririchnium magnificum*

Caririchnium leonardii	*Hadrosauropodus leonardii*
Caririchnium lotus	*Caririchnium lotus*
Caririchnium Kyoungsookimi	*Hadrosauropodus Kyoungsookimi*
Hadrosauropodus langstoni	*Hadrosauropodus langstoni*
Iguanodontipus burreyi	*Iguanodontipus burreyi*
Iguanodontipus billsarjeanti	*Caririchnium billsarjeanti*

Notably, the aforementioned classification schemes require follow-up studies to manually identify and classify all ichnotaxon diagnostics. Thus, ichnotaxon oversplitting problems can still occur because of subjective measures of outlining and experience. Therefore, this study attempted to automatically classify ornithopod dinosaur tracks using convolutional neural networks to circumvent these problems and to distinguish between validated ichnotaxa (i.e., *Caririchnium*, *Hadrosauropodus*, and *Iguanodontipus*).

Fig 1. Changes in large ornithopod ichnotaxa classification since 2004. The scale bar is 10 cm.

Methods

Convolution neural network and transfer learning

We developed a machine learning model for classifying the outline images of large ornithopod dinosaur footprints using a convolutional neural network (CNN). Since the first appearance of CNN for image classification [31], many researchers have produced various CNN models, such as LeNet [32], VCG-Net [33], GoogleNet [34], and ResNet [35]. Such CNNs generally comprise multiple layers, and each layer performs specific functions in the network as follows [36]. A convolution layer extracts features from the input image and produces a 2D activation map that exhibits features detected at a given location in the input image [31]. A nonlinearity layer introduces nonlinearities into the network. The pooling layer reduces the number of parameters in the network by reducing the spatial resolution of the feature maps. Finally, a fully connected layer is used to generate the final prediction from the feature maps produced by the previous layers.

Among the different variants of CNNs, we utilized Xception [37] to analyze footprint outlines of large ornithopod dinosaurs. Typical convolutions in CNNs operate on each feature map (i.e., channel) independently of the input (i.e., depthwise convolution) or the entire feature map (i.e., pointwise convolution). In Xception, as an alternative to these convolutions, a depth-wise separable convolution is introduced for computational efficiency, which operates on each channel first with depth-wise convolution and then applies point-wise convolution to increase the number of channels in the output. Xception has 14 modules and 36 convolution layers (Fig 2) and is a lightweight model of Inception V3 [37]. However, Xception differs from Inception v3 because it

prevents the loss of information by performing depthwise separable convolution and removing nonlinear functions from the operation [37].

Although Xception is known for its good performance and computational efficiency [37], our new machine learning model is inherently constrained by the small number of available ornithopod dinosaur footprint illustrations. To circumvent this problem, we employed transfer learning to improve the performance of the new model [38] by utilizing Xception as a feature extractor and weights trained using ImageNET. ImageNET weights were employed to enhance the learning performance and accuracy of the machine learning model. Instead of the original fully connected layer, our model incorporated a classification layer into a network consisting of two dense layers, one with 64 and 32 nodes weighted by the Rectified Linear Unit (ReLU) function and the other with three nodes weighted by the softmax function (Fig 2). The answer labels were *Caririchnium*, *Hadrosauropodus*, and *Iguanodontipus* (Fig 1).

To avoid any overfitting errors owing to the small training datasets, we also applied an L2 regulation of 0.001 for weight attenuation and a dropout layer to temporarily remove random nodes from the network [39]. As an optimizer, Adam (Adaptive Moment Estimation) [40] was implemented to the transfer learning with the learning rate of 1×10^{-6}.

Fig 2. Schematic architecture of the machine learning model developed for large ornithopod ichnotaxa classification in this study.

Data collection and preprocessing

Following the nomenclature proposed by Díaz-Martínez et al. [3], we collected 274 dinosaur footprint illustrations from the literature: 111 *Caririchnium* [8–10,21,24,29,41–44], 36 *Hadrosauropodus* [2,14,41,45–50], and 127 *Iguanodontipus* [13,51–53]. The geographical distributions of these footprints are listed in Table 2. The collected illustrations (i.e., images) were redrawn using Adobe Illustrator to precondition the image data. For example, unnecessary internal structures and forepaw data were removed (Fig 3) because the ichnotaxonomy of the large ornithopod dinosaur footprints was revised based only on the contours of the hind feet [3]. In this study, we used only pes outlines. In addition, all image data in the training set were flipped horizontally and stored individually. This process is introduced to generate opposite footprints for each track.

The machine learning model developed in this study is inherently subject to a relatively small amount of dinosaur footprint data. To prevent overfitting, we utilized the ImageDataGenerator for random transformation and normalization of the given image datasets, which is a class in the Keras deep learning library [54]. Data augmentation with the ImageDataGenerator was implemented by rescaling, rotating (< 10°), shifting width (< 10%), height (< 10%), and zooming (< 20%), which operated randomly on the preconditioned image dataset. Shearing the input images in the ImageDataGenerator was not used in this step to preserve the characteristics of the dinosaur footprints (e.g., bilobed heel).

To classify the outline images of the large ornithopod dinosaur tracks effectively, the input images for the machine learning model developed in this study were prepared as follows: Footprint images were prepared to show single black-lined pes with no internal structures or manus (Fig 3). The line

thickness of the pes outline did not considerably affect the performance of the model when the features were recognized. The footprint image was placed on a white square background with the toe length occupying at least 66% of the background length. In this study, we used a 500×500 pixel white background with a line thickness of 9 pt. Finally, the center of the footprint was placed at the center of the background, with the middle toe pointing upward. Thus, we executed a robust feature extraction operation on the input footprint images of large ornithopod dinosaurs.

Fig 3. Examples of redrawn footprint illustrations. The solid lines represent the outlines of the original footprints included in the train dataset, whereas the dotted lines represent the excluded outlines. A, *Amblydactylus kortmeyeri* [42]; B, *Iguanodontipus billsarjeanti* [8]; C, *Caririchnium* [41]; D, *Caririchnium lotus* [44]; E, *Caririchnium magnificum* [29]; F, *Caririchnium* [47]; G, *Caririchnium kyoungsookimi* [50]; H, *Caririchnium* [29]; I–J, *Hadrosauropodus langstoni* [2]; K, *Iguanodontipus burreyi* [53]; L, *Iguanodontipus* [52]; M, *Iguanodontipus* [6]; N–O, *Iguanodontipus* [52]. The scale bar is 10 cm and [6] is not included in the training dataset.

Table 2. Geographic distribution of the large ornithopod dinosaur track data used in this study.

	Caririchnium	*Hadrosauropodus*	*Iguanodontipus*
North America	3	16	x

South America	4	1	x
Asia	101	6	x
Europe	3	13	127

Results and Discussion

We developed a machine learning model to classify the ichnogenera of large ornithopod dinosaur footprint illustrations according to the ichnotaxon criteria presented by Díaz-Martínez et al. [3]. The machine learning model was trained for 162 epochs using 274 data images, excluding the horizontally flipped images. The training procedures were stopped after 100 more epochs, after which the validation loss function did not improve (Fig 4). The accuracy and loss functions of the machine-learning model tended to saturate at relatively early epochs, indicating that the model had already learned all it could from the given training data. The final accuracy was 96.36% and the validation accuracy was 92.59%. The trained machine-learning model provided a list of classification probabilities for a given footprint image with respect to the labels *Iguanodontipus*, *Caririchnium*, and *Hadrosauropodus*.

In the following section, we demonstrate the performance of the machine learning model with footprint illustrations that were not included in the training dataset (Fig 5).

Fig 4. Changes of accuracy (A) and loss (B) functions of the machine learning model, with the training (red solid) and validation (blue dashed) datasets. In both trends, the model becomes stable at relatively early epochs.

Fig 5. Test result of large ornithopod tracks classification model. A, *Caririchnium* from Uiesung, Republic of Korea [16]; B, High quality illustration of *Caririchnium* from Gunbuk, Republic of Korea [26]; C, Low quality illustration of *Caririchnium* from Gunbuk, Republic of Korea [26]; D, *Hadrosauropodus* from La Llau de la Costa, Spain [51]; E, *Hadrosauropodus* from Tetori, Japan [55]; F, *Therangospodus oncalensis* from Tierras Amigas, Spain [13]; G, *Ornithopodichnus* from Sichuan, China [58]; H, *Iguanodontipus* track from Soria, Spain [52] (the footprint is illustrated with multiple lines); I, Ornithopod footprint from Qijiang, China [10].

Caririchnium - Uiesung, Republic of Korea

The dinosaur footprint tracks at Mancheon-ri, Uisung, and Gyeongsangbuk-do in South Korea were reported to be from *Caririchnium* [16]. Although some *Caririchnium* tracks have manus, they were removed during the preconditioning of the image data. The machine learning model classified it as *Caririchnium* with a probability of 97.32% (Fig 5A).

Caririchnium - Gunbuk, Republic of Korea

The dinosaur footprint tracks at Gunbuk-myeon, Gyeongsangbuk-do, South Korea, have been reported to be from *Caririchnium* [26]. In this example, the original image from the given literature had a low-quality footprint image. The machine learning model classified the footprint as *Iguanodontipus* with a probability of 61.66%, by estimating the lowest classification probability of 17.74% for the *Caririchnium* label (Fig 5C). However, when we enhanced the input image, as shown in Fig 5B, the model classified the footprint as *Iguanodontipus* with a probability of 99.48% and the lowest probability of 0.03% for *Caririchnium*. This discrepancy between the literature [26] and the prediction might result from the input image showing an elongated and narrow digit impression of *Iguanodontipus*, rather than a short, wide digit impression of *Caririchnium*.

Hadrosauropodus **- La Llau de la Costa locality, Spain**

The dinosaur footprints found in the Tremp Formation, Spain, were reported as *Hadrosauropodus* [51]. Among the reported tracks, sample MCD-5142, identified in La Llau de la Costa, was used (Fig 5D). The model classified the given input footprint image as *Hadrosauropodus* with a probability of 88.12%.

Hadrosauropodus **- Tetori, Japan**

The dinosaur footprint (TGUSE-DT1007) found at Tetori, Japan, was reported as being from *Caririchnium* because its digits became slightly narrower distally but remained blunt [55]. However, the machine learning model classified the given footprint (Fig 5E) as *Hadrosauropodus* with a 91.35% probability. Because the given footprint has a bilobed heel, the model classified the

input image as *Hadrosauropodus* rather than *Caririchnium*.

Iguanodontipus - Tierras Amigas, Spain

The footprints of *Therangospodus oncalensis* in Tierras Amigas, Spain, were initially reported as theropods and then reinterpreted as the ornithopod *Iguanodontipus oncalensis* [13]. We used samples identified at the Salgar de Sillas site (Fig 5F). The model classified the input footprint as *Iguanodontipus* with a probability of 99.86%.

Ornithopodichnus - Sichuan, China

The footprints found in Sichuan, China, were initially classified as *Ornithopodichnus* [58] and were later invalidated by Díaz-Martínez et al. [3]. The machine learning model showed that the given footprint (Fig 5G) could be classified as *Iguanodontipus* with a 90.85% probability.

Iguanodontipus - Las Cuestas I, Spain

The ornithopod dinosaur footprints found at Las Cuestas I, Spain, were reported as being from *Iguanodontipus* [52], and the initial classification was reconfirmed by Díaz-Martínez et al. [3]. In this example, the footprint illustration is composed of multiple lines on trackway STC-1 (Fig 5H). The model classified the input footprint as *Iguanodontipus* with a probability of 99.23%.

Ornithopod footprint - Qijiang, China

Numerous ornithopod footprints have been reported for the *Caririchnium lotus* fossil site in Qijiang, China, which is enclosed within the Cretaceous Jiaguan Formation [10]. Although the distribution of fossil tracks was mapped for the entire site, some footprints were poorly preserved; hence, the illustrations did not show the distinctive morphological characteristics of the considered footprints, as shown in Fig 5I. Although the footprint was taken from the trackway of the *Caririchnium lotus*, the model classified it as *Iguanodontipus* with a probability of 75.48%. This indicates that the classification probability tends to be inaccurate for poorly preserved fossil tracks.

This large ornithological dinosaur footprint illustration classification model showed over 92% learning and validation accuracy. Machine learning models are expected to make researchers' judgments regarding ornithopod dinosaur footprints more reasonable and sophisticated. The machine-learning model will likely prevent erroneous naming of the large ornithopod dinosaur footprint ichnotaxa and act as a precaution against the oversplitting problem.

From the testing results listed above, we found that the machine learning model developed in this study was properly trained to classify any given footprint illustration data for large ornithopod dinosaurs. For example, although the *Hadrosauropodus* footprints reported in Tetori, Japan [55] were not included in the training data, the model classified the given data with a high probability. However, the *Caririchnium* footprint reported in Gunbuk, Republic of Korea [26] showed a substantially different classification result between the model and literature. As the Gunbuk footprint has an elongated and narrow digit impression (Fig 5B), the classification results appear to be consistent with the standards for large ornithopod ichnotaxa [3].

However, the performance of our machine-learning model is inherently dependent on the quality

of the footprint illustration data. For example, the classification probabilities for the footprint data shown in Figs 5B and 5C were estimated differently because of the image quality, although the classification was identical for both cases. Furthermore, the poorly preserved track illustration (Fig 5I) shows results similar to the classification probabilities of the low-quality images (Fig 5C). These discrepancies may be because the model was trained primarily with high-quality data. However, the model was also capable of classifying footprint illustrations with multiple outlines (Fig 5H). This indicates that excessively thick or thin footprint outlines would reduce the classification performance of this model because such lines can hinder feature extraction in the model. Therefore, the model performs better with well-preserved footprints.

Because the machine-learning model developed in this study is a typical supervised learning model, it is impossible to introduce a new label or class. For example, the *Ornithopodichnus* reported in Sichuan, China [56], was excluded from the ornithopod ichnogenera by Díaz-Martínez et al. [3]. Therefore, the labels used to train the model did not include *Ornithopodichnus* [56, 57]. If the reference ichnogenus [3] needs to be revised or a new ichnogenus introduced, the model must be retrained with the footprint datasets available at that time.

In addition, our machine learning model could not correctly classify theropod and sauropod footprints. Therefore, it is essential for researchers to carefully select the ornithopod footprints before executing the model. Careful attention is required to distinguish the footprints of ornithopods and theropods. This limitation can be resolved by expanding the database to include both theropod and sauropod footprints [58].

In summary, we developed a machine-learning model to classify large ornithological dinosaur footprints with an overall learning and validation accuracy of 92%. Although this study used

illustrations rather than photos or 3D data, it is the first to apply machine learning techniques at the academic level to dinosaur footprints. Furthermore, the machine learning model will likely prevent misnaming of large ornithopod dinosaur footprint ichnotaxa, thereby safeguarding against the oversplitting problem.

Acknowledgements

This study was supported by the National Research Foundation of Korea (NRF-2021R1A4A5026233 and NRF-2021R1A2C1012030).

References

1. Min H, Koo Geun H. Dinosaur footprints from the Cretaceous of South Korea: with reference to Cheollanam-do dinosaur sites. The Paleontological Communications Society of Korea; 2004.

2. Lockley MG, Nadon G, Currie PJ. A diverse dinosaur-bird footprint assemblage from the Lance Formation, Upper Cretaceous, eastern Wyoming: Implications for ichnotaxonomy. Ichnos. 2004;11: 229-249. doi: 10.1080/10420940490428625.

3. Díaz-Martínez I, Pereda-Suberbiola X, Pérez-Lorente F, Canudo JI. Ichnotaxonomic review of large ornithopod dinosaur tracks: Temporal and geographic implications. PLOS ONE. 2015;10: e0115477. doi: 10.1371/journal.pone.0115477.

4. Lockley MG, Xing L, Lockwood JAF, Pond S. A review of large Cretaceous ornithopod tracks, with special reference to their ichnotaxonomy. Biol J Linn Soc Lond. 2014;113: 721-736. doi: 10.1111/bij.12294.

5. Lucas S, Sullivan R, Jasinski S, Ford T. Hadrosaur footprints from the Upper Cretaceous Fruitland Formation. New Mexico: San Juan Basin, and the ichnotaxonomy of large ornithopod footprints. New Mexico Museum of Natural History bulletin; 2011. p. 53.

6. Sarjeant WAS, Delair JB, Lockley MG. The footprints of iguanodon: A history and taxonomic study. Ichnos. 1998;6: 183-202. doi: 10.1080/10420949809386448.

7. Molina MMR, Lorente FP, Carrera PR. Análisis de la parataxonomía utilizada con las huellas de dinosaurio. Universidad de La Rioja; 2003.

8. Meyer C, Thüring B. The first iguanodontid dinosaur tracks from the Swiss Alps (Schrattenkalk Formation, Aptian). Ichnos. 2003;10: 221–228. doi: 10.1080/10420940390256186.

9. Xing L, Lockley MG, Marty D, Klein H, Yang G, Zhang J, et al. A diverse saurischian (theropod–sauropod) dominated footprint assemblage from the Lower Cretaceous Jiaguan Formation in the Sichuan Basin, southwestern China: A new ornithischian ichnotaxon, pterosaur tracks and an unusual sauropod walking pattern. Cret Res. 2016;60: 176-193. doi: 10.1016/j.cretres.2015.12.005.

10. Xing L, Lockley MG, Marty D, Zhang J, Wang Y, Klein H, et al. An ornithopod-dominated tracksite from the Lower Cretaceous Jiaguan Formation (Barremian–Albian) of Qijiang, south-central China: New discoveries, ichnotaxonomy, preservation and palaeoecology. PLOS ONE. 2015;10: e0141059. doi: 10.1371/journal.pone.0141059.

11. Xing L, Lockley MG, Zhang J, Klein H, Marty D, Peng G, et al. The longest theropod trackway from East Asia, and a diverse sauropod-, theropod-, and ornithopod-track assemblage from the Lower Cretaceous Jiaguan Formation, southwest China. Cret Res. 2015;56: 345-362. doi: 10.1016/j.cretres.2015.05.008.

12. Castanera D, Bádenas B, Aurell M, Canudo JI, Gasca JM. New ornithopod tracks from the Lower Cretaceous El Castellar Formation (Spain): Implications for track preservation and evolution of ornithopod footprints. Palaeogeogr Palaeoclimatol Palaeoecol. 2022;591: 110866. doi: 10.1016/j.palaeo.2022.110866.

13. Castanera D, Pascual C, Razzolini NL, Vila B, Barco JL, Canudo JI. Discriminating

between medium-sized tridactyl trackmakers: Tracking ornithopod tracks in the base of the Cretaceous (Berriasian, Spain). PLOS ONE. 2013;8: e81830. doi: 10.1371/journal.pone.0081830.

14. Enriquez NJ, Campione NE, White MA, Fanti F, Sissons RL, Sullivan C, et al. The dinosaur tracks of Tyrants Aisle: An Upper Cretaceous ichnofauna from Unit 4 of the Wapiti Formation (Upper Campanian), Alberta, Canada. PLOS ONE. 2022;17: e0262824. doi: 10.1371/journal.pone.0262824.

15. Kim HJ, Hwang KG, Jeong HH, Ahn HY, Choi CU, Lim HS, et al. Dinosaur track-bearing deposits in Sagok Formation (Cretaceous) at Sinseong-ri, Cheonsong-gun, Korea: Occurrences and paleoenvironments. J Geol Soc Korea. 2019;55: 495-511. doi: 10.14770/jgsk.2019.55.5.495.

16. Lee J-H, Lockley MG, Jung S, Ha Y, Cho KR, Lim JD, et al. Dinosaur tracksites from the Cretaceous Sagok Formation in the Uiseong Regional Geopark, South Korea: Historical, palaeobiological perspectives. Hist Biol. 2022: 1-9. doi: 10.1080/08912963.2022.2106563.

17. Melchor RN, Rivarola DL, Umazano AM, Nalín Moyano M, Mendoza Belmontes FR. Elusive Cretaceous Gondwanan theropods: The footprint evidence from central Argentina. Cret Res. 2019;97: 125-142. doi: 10.1016/j.cretres.2019.01.004.

18. Oukassou M, Klein H, Lagnaoui A, Charrière A, Saber H, Gierliński GD, et al. Polyonyx-like tracks from Middle-?Upper Jurassic red beds of Morocco: Implications for sauropod communities on southern margins of tethys. Palaeogeogr Palaeoclimatol Palaeoecol. 2019;536: 109394. doi: 10.1016/j.palaeo.2019.109394.

19. Romilio A, Salisbury SW. Large dinosaurian tracks from the Upper Cretaceous (Cenomanian–Turonian) portion of the Winton Formation, Lark Quarry, central-western Queensland, Australia: 3D photogrammetric analysis renders the ‘stampede trigger’ scenario unlikely. Cret Res. 2014;51: 186-207. doi: 10.1016/j.cretres.2014.06.003.

20. Tsukiji Y, Azuma Y, Shiraishi F, Shibata M. A diverse theropod footprint assemblage from the Lower Cretaceous Kitadani Formation, Tetori Group, central Japan. Cret Res. 2019;97: 16-33. doi: 10.1016/j.cretres.2019.01.003.

21. Tsukiji Y, Azuma Y, Shiraishi F, Shibata M, Noda Y. New ornithopod footprints from the Lower Cretaceous Kitadani Formation, Fukui, Japan: Ichnotaxonomical implications. Cret Res. 2018;84: 501-514. doi: 10.1016/j.cretres.2017.12.011.

22. Xing L-D, Ba J, Lockley MG, Klein H, Yan S-W, Romilio A, et al. Late Triassic sauropodomorph and Middle Jurassic theropod tracks from the Xichang Basin, Sichuan Province, southwestern China: First report of the ichnogenus Carmelopodus. J Palaeogeogr. 2018;7: 1-13. doi: 10.1016/j.jop.2017.11.004.

23. Xing L, Lockley MG, Mao Z, Klein H, Gu Z, Bai C, et al. A new dinosaur track site from the earliest Cretaceous (Berriasian) part of the Tuchengzi Formation, Hebei Province, China: Implications for morphology, ontogeny and paleocommunity structure. Palaeogeogr Palaeoclimatol Palaeoecol. 2021;580: 110619. doi: 10.1016/j.palaeo.2021.110619.

24. Xing L, Lockley MG, Romilio A, Klein H, Zhang J, Chen H, et al. Diverse sauropod-theropod-dominated track assemblage from the Lower Cretaceous Dasheng Group of

Eastern China: Testing the use of drones in footprint documentation. Cret Res. 2018;84: 588-599. doi: 10.1016/j.cretres.2017.12.012.

25. Xing L, Wang Y, Lockley MG, Li M, Gao L, Yin Z, et al. Theropod and other dinosaurian tracks from the upper Lower Cretaceous of northeastern China: Overlooked Fuxin Biota records reinvestigated. Cret Res. 2022;135: 105190. doi: 10.1016/j.cretres.2022.105190.

26. Yoon HS, Lee Y-N, Jung S-H, Kong D-Y, Kim S-H, Son M. A juvenile ornithopod tracksite from the Lower Cretaceous Haman Formation, South Korea. Cret Res. 2021;125: 104877. doi: 10.1016/j.cretres.2021.104877.

27. Lallensack JN. Automatic generation of objective footprint outlines. PeerJ. 2019;7: e7203. doi: 10.7717/peerj.7203.

28. Sternberg CM. Dinosaur tracks from Peace River, British Columbia. Annual report of the national museum of Canada1932.

29. Leonardi G. Le impronte fossili di dinosauri (with the holotypes of the ichnogen. Caririchnium and Ligabueichnium). 1984. p. 163-186.

30. Lucas SG. Tetrapod footprint biostratigraphy and biochronology. Ichnos. 2007;14: 5-38. doi: 10.1080/10420940601006792.

31. LeCun Y, Boser B, Denker JS, Henderson D, Howard RE, Hubbard W, et al. Backpropagation applied to handwritten ZIP code recognition. Neural Comput. 1989;1: 541-551. doi: 10.1162/neco.1989.1.4.541.

32. Lecun Y, Bottou L, Bengio Y, Haffner P. Gradient-based learning applied to document

recognition. Proc IEEE. 1998;86: 2278-2324. doi: 10.1109/5.726791.

33. Simonyan K, Zisserman A. Very deep convolutional networks for large-scale image recognition. Clin Orthop Relat Res. 2015;abs/1409.1556.

34. Szegedy C, Wei L, Yangqing J, Sermanet P, Reed S, Anguelov D, et al., editors. Going deeper with convolutions IEEE Conference on Computer Vision and Pattern Recognition (CVPR); 2015; 2015. doi: 10.1109/CVPR.2015.7298594.

35. He K, Zhang X, Ren S, Sun J, editors. Deep residual learning for image recognition IEEE Conference on Computer Vision and Pattern Recognition (CVPR); 2016; 2016. doi: 10.1109/CVPR.2016.90.

36. Albawi S, Mohammed TA, Al-Zawi S, editors. Understanding of a convolutional neural network International Conference on Engineering and Technology (ICE T); 2017; 2017. doi: 10.1109/ICEngTechnol.2017.8308186.

37. Chollet F, editor. Xception: Deep learning with depthwise separable convolutions. In: Proceedings of the IEEE conference on computer vision and pattern recognition; 2017: 1800–1807. doi: 10.1109/CVPR.2017.195.

38. Torrey L, Shavlik J. Transfer learning. Handbook of research on machine learning applications and trends: Algorithms, methods, and techniques. IGI Global; 2010. pp. 242-264.

39. Srivastava N, Hinton G, Krizhevsky A, Sutskever I, Salakhutdinov R. Dropout: A simple way to prevent neural networks from overfitting. J Mach Learn Res. 2014;15: 1929–1958.

40. Kingma DP, Adam BJ. A method for stochastic optimization. Clin Orthop Relat Res. 2015;abs/1412.6980.

41. Huh M, Hwang KG, Paik IS, Chung CH, Kim BS. Dinosaur tracks from the Cretaceous of South Korea: Distribution, occurrences and paleobiological significance. Isl Arc. 2003;12: 132-144. doi: 10.1046/j.1440-1738.2003.00386.x.

42. Currie PJ, Sarjeant WAS. Lower Cretaceous dinosaur footprints from the peace River Canyon, British Columbia, Canada. Palaeogeogr Palaeoclimatol Palaeoecol. 1979;28: 103-115. doi: 10.1016/0031-0182(79)90114-7.

43. Kim JY, Lockley MG, Chun HY. New dinosaur tracks from the Lower Cretaceous (Valanginian-Hauterivian) Saniri Formation of Yeongdong area, central Korea: Implications for quadrupedal ornithopod locomotion. Cret Res. 2016;61: 5-16. doi: 10.1016/j.cretres.2015.12.023.

44. Xing L, Wang F, Pan S, Chen W. The discovery of dinosaur footprints from the Middle Cretaceous Jiaguan Formation of Qijiang County, Chongqing City. Acta Geol Sin. 2007;81: 1591-1602.

45. McCrea R, Buckley L, Currie P, Plint G, Haggart J, Helm C, et al. A review of vertebrate track-bearing formations from the Mesozoic and earliest Cenozoic of western Canada with a description of a new theropod ichnospecies and reassignment of an avian ichnogenus. New Mexico Museum of Natural History and Science bulletin; 2014. pp. 5-93.

46. Lockley MG. Dinosaur footprints from the Dakota Group of eastern Colorado. Mt Geol. 1987.

47. Currie PJ, Nadon GC, Lockley MG. Dinosaur footprints with skin impressions from the Cretaceous of Alberta and Colorado. Can J Earth Sci. 1991;28: 102-115. doi: 10.1139/e91-009.

48. Díaz-Martínez I, de Valais S, Cónsole-Gonella C. First evidence of Hadrosauropodus in Gondwana (Yacoraite Formation, Maastrichtian-Danian), northwestern Argentina. J Afr Earth Sci. 2016;122: 79-87. doi: 10.1016/j.jafrearsci.2016.02.012.

49. Vila B, OMS O, Fondevilla V, Gaete R, Galobart À, Riera V, et al. The Latest Succession of Dinosaur Tracksites in Europe: Hadrosaur Ichnology, Track Production and Palaeoenvironments. PLOS ONE. 2013;8(9):e72579.

50. Lim J-D, Lockley MG, Kong D-Y. The trackway of a quadrupedal ornithopod from the Jindong Formation (Cretaceous) of Korea. Ichnos. 2012;19: 101-104. doi: 10.1080/10420940.2012.664059.

51. Lockwood JAF, Lockley MG, Pond S. A review of footprints from the Wessex Formation (Wealden Group, Lower Cretaceous) at Hanover Point, the Isle of Wight, southern England. Biol J Linn Soc Lond. 2014;113: 707-720. doi: 10.1111/bij.12349.

52. Pascual-Arribas C, Hernández-Medrano N, Latorre-Macarrón P, Sanz-Pérez E. El icnogenero Iguanodontipus en el yacimiento de 'Las Cuestas I' (Santa Cruz de Yanguas, Soria, España). Stud Geol Salmanticensia. 2010;46: 106.

53. Beckles SH. On the ornithoidichnites of the wealden. Q J Geol Soc. 1854;10: 456-464. doi: 10.1144/GSL.JGS.1854.010.01-02.52.

54. Chollet F. Building powerful image classification models using very little data. Keras blog; 5; 2016.

55. Matsukawa M, Shibata K, Kukihara R, Koarai K, Lockley MG. Review of Japanese dinosaur track localities: Implications for ichnotaxonomy, paleogeography and stratigraphic correlation. Ichnos. 2005;12: 201-222. doi: 10.1080/10420940591009231.

56. Xing L, Lockley MG. First report of small Ornithopodichnus trackways from the Lower Cretaceous of Sichuan, China. Ichnos. 2014;21: 213-222. doi: 10.1080/10420940.2014.951998.

57. Kim JY, Lockley MG, Kim HM, Lim J-D, Kim KS. New dinosaur tracks from Korea, Ornithopodichnus masanensis ichnogen. et ichnosp. nov. (Jindong Formation, Lower Cretaceous): Implications for polarities in ornithopod foot morphology. Cret Res. 2009;30: 1387-1397. doi: 10.1016/j.cretres.2009.08.003.

58. Lallensack JN, Romilio A, Falkingham PL. A machine learning approach for the discrimination of theropod and ornithischian dinosaur tracks. J R Soc Interface. 2022;19: 20220588. doi: 10.1098/rsif.2022.0588.

Click here to access/download;Figure;Fig1.tiff

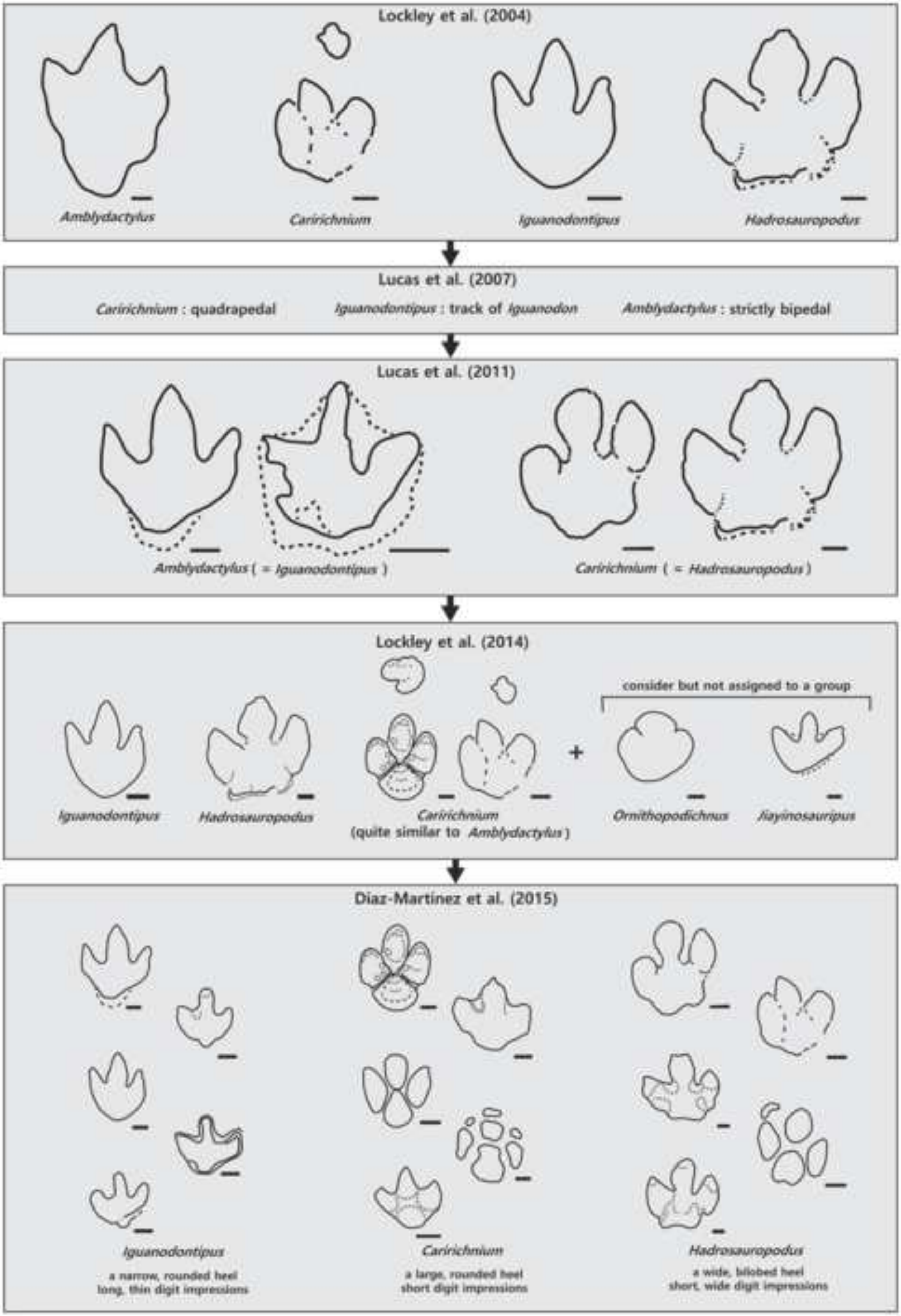

Fig 02

Click here to access/download;Figure;Fig2.tif

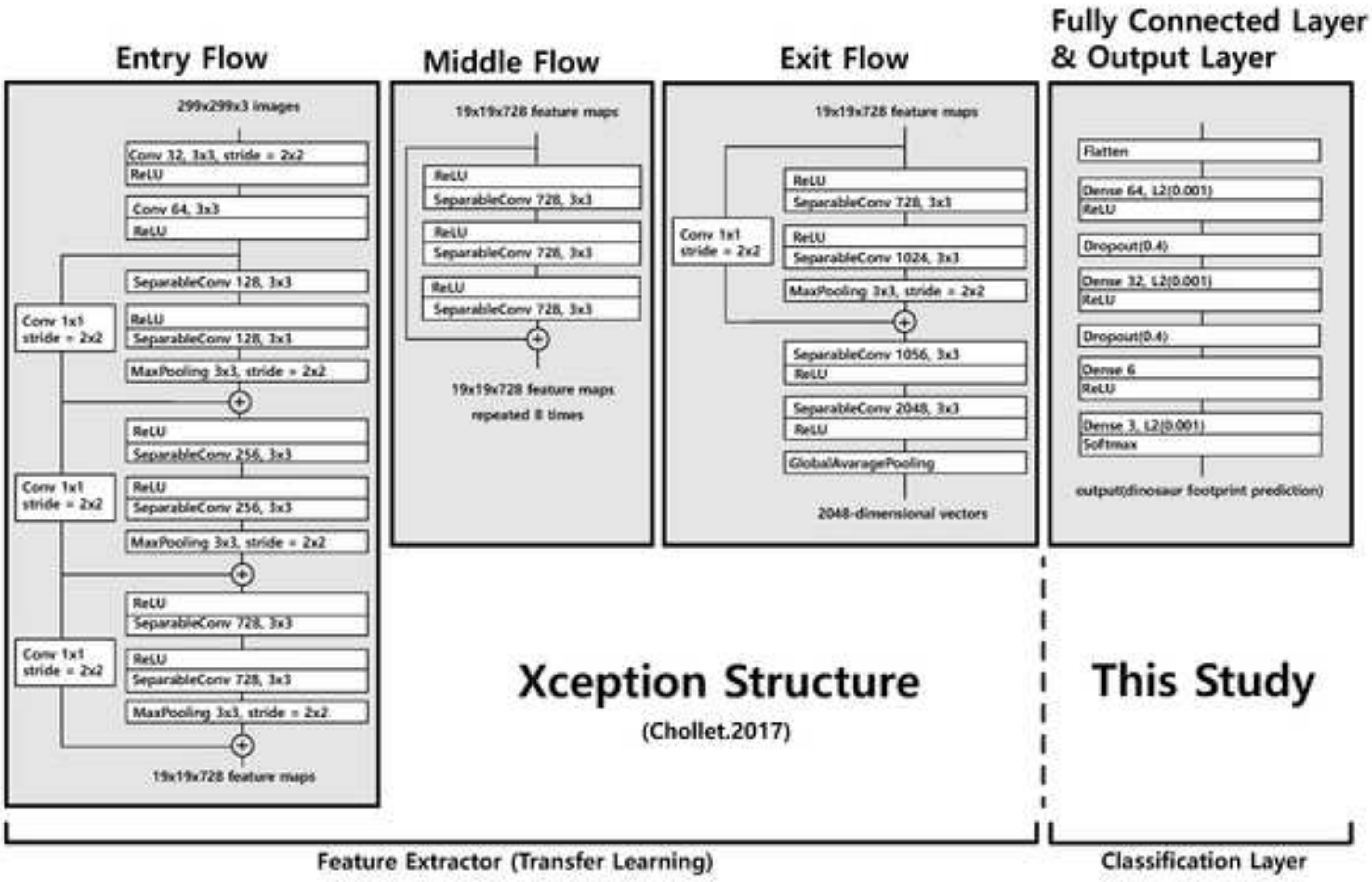

Click here to access/download;Figure;Fig3.tif

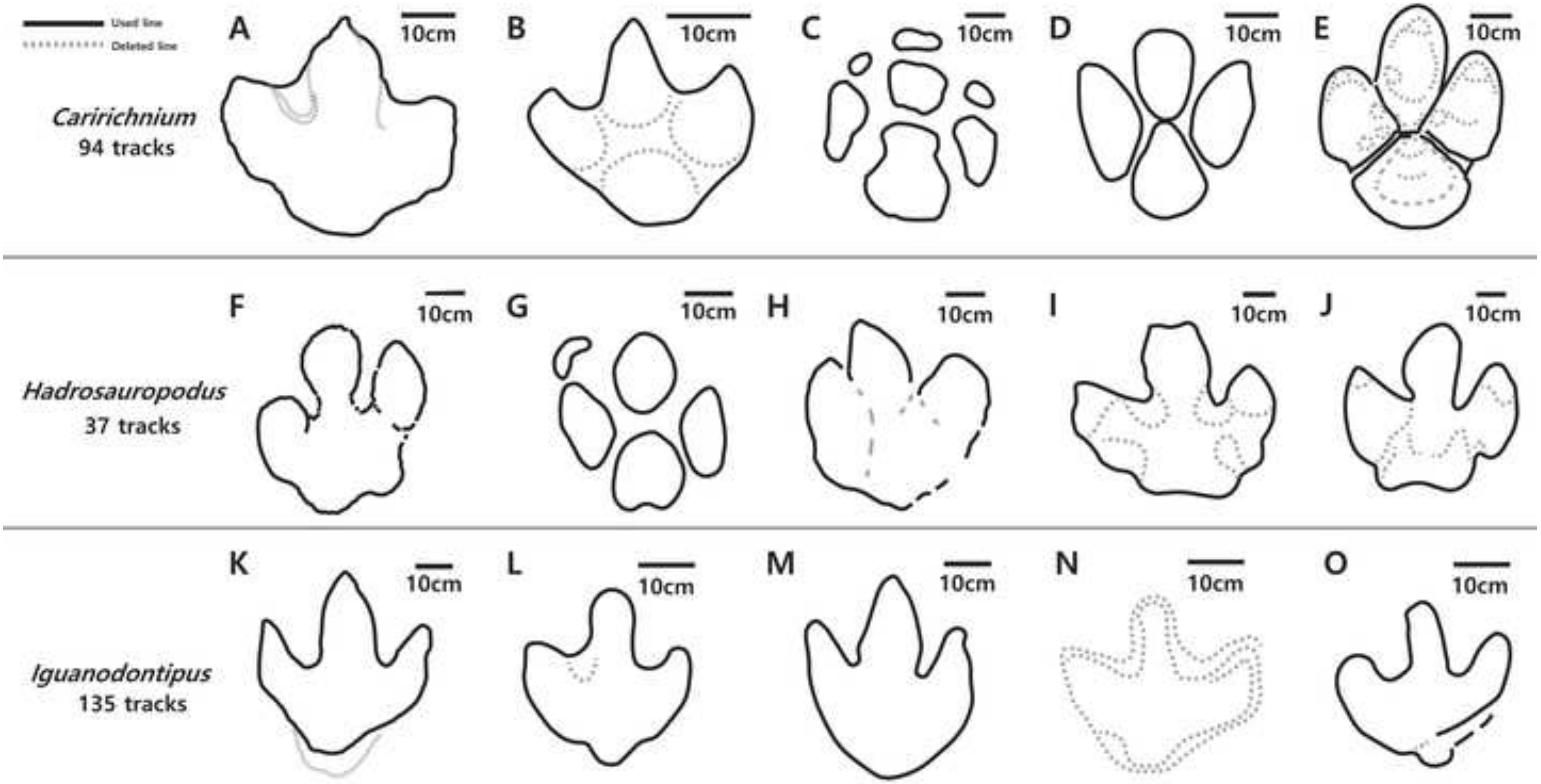

Fig 04

Click here to access/download;Figure;Fig4.tif

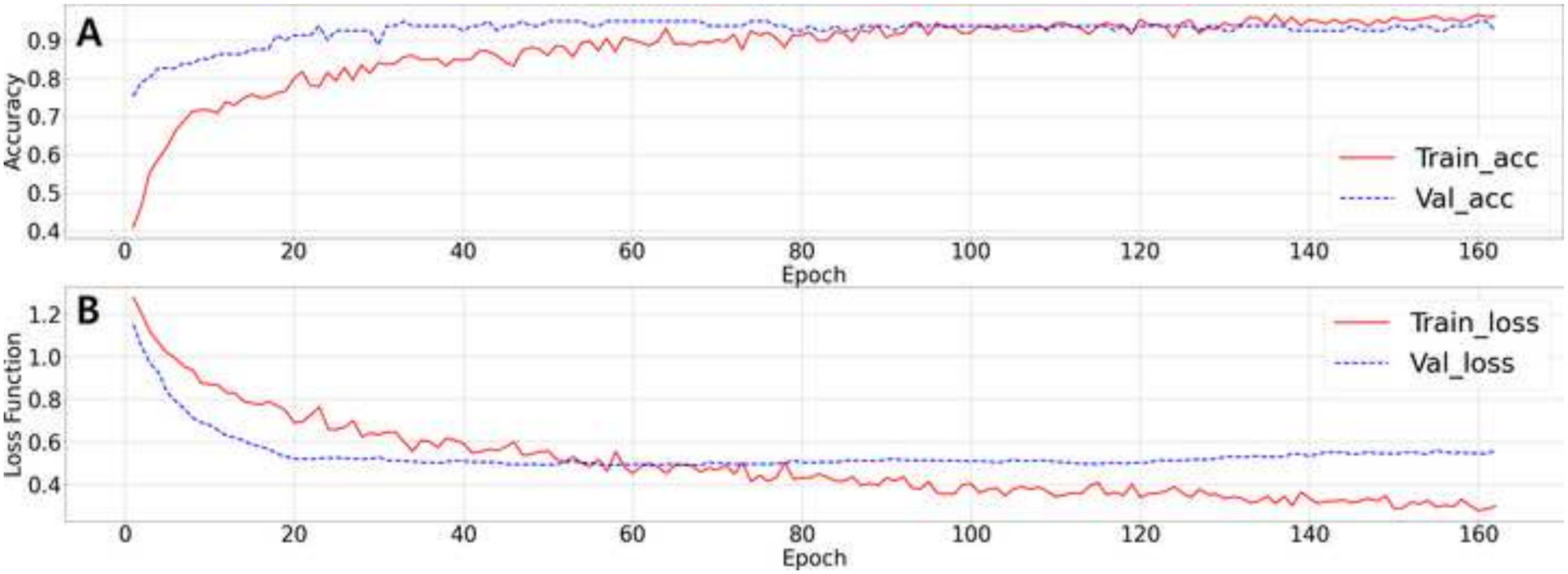

Fig 05

Click here to access/download;Figure;Fig5.tif

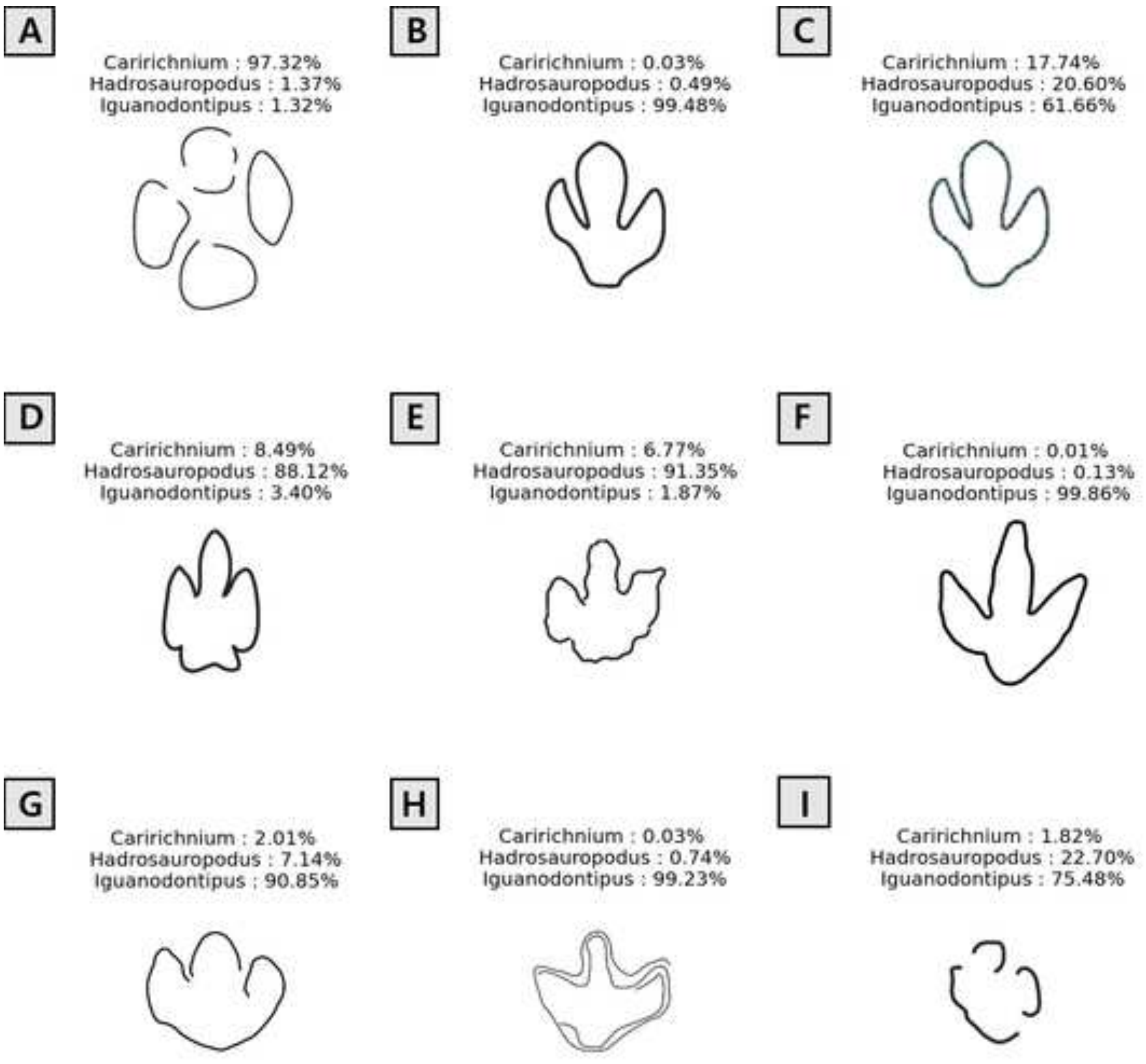

Supplement: Attachment — Submitted filename: PONE-D-23-08114_reviewer.pdf [file pone.0293020.s001.pdf]
