# Supplementary material for: Classification of large ornithopod dinosaur footprints using Xception transfer learning
Source: PLoS One. 2023 Nov 29;18(11):e0293020. doi: 10.1371/journal.pone.0293020 (PMC10686485; doi:10.1371/journal.pone.0293020)
Supplement: Attachment — Submitted filename: Rebuttal_letter.docx [file pone.0293020.s002.docx]

For your information, our detailed responses to the reviewers’ comments are provided in blue.

*Editor Comments:*

*Thank you for submitting your work for publication in PLoS One.
I kindly ask you to refer to the reviewers’ suggestions in the minor revision.*

- We were very delighted to hear that our paper was mostly satisfied by the reviewers. We closely followed the reviewers’ comments closely when revising the manuscript.

*Reviewer #1: Reviewer Summary:
The paper titled "Classification of Large Ornithopod Dinosaur Footprints Using Xception Transfer Learning" introduces a novel approach to tackle oversplitting challenges in the classification of large ornithopod dinosaur footprints. The authors adopt a convolutional neural network-based Xception transfer learning technique to automate the classification process and demonstrate that remarkable accuracy can be attained even with a relatively small supervised image training set (less than 300 images).

Based on my evaluation, I firmly believe that this paper deserves publication (once revisions have been completed) primarily due to its implementation of a machine learning model, which represents a positive and promising step forward in the field of dinosaur ichnological studies. The utilization of advanced computational techniques holds substantial potential in enhancing the accuracy and efficiency of footprint classification, thus contributing to the advancement of this scientific domain.*

- We appreciate your constructive comments on our submission. We closely followed your comments closely when revising the manuscript.

*My main concern revolves around the central question addressed by the authors in the current version of the paper: the oversplitting of large ornithopod dinosaur footprints. It is important to clarify that the primary objective of the paper is not to comprehensively resolve the oversplitting issue, but rather to validate the findings of Díaz-Martínez et al. (2015). This clarification does not diminish the merit of the paper; however, it explains the reliance on the findings of Díaz-Martínez et al. (2015) and the inclusion of only three ichnogenera (Caririchnium, Hadrosauropodus, and Iguanodontipus) in the training set. Consequently, the evaluation of test images is naturally limited to these three ichnotaxa. This focus does not diminish the merit of the paper but rather underscores the strength of their method in verifying ichnotaxonic assignments, albeit based on outlines.*

- Agreed. We clarified the primary objective of our study as “it is the first application of machine-learning techniques at the academic level for verifying the ichnotaxonic assignments of large ornithopod dinosaur footprints.” (see Abstract). Additionally, we state “In this study, we developed a machine learning classification method for large ornithopod dinosaur footprints (Table 1) based on the classification criteria of Díaz-Martínez et al. [3].” in Introduction.

*Additionally, in the abstract, the authors claim that their approach represents the first application of machine-learning techniques at the academic level for large ornithopod dinosaur footprints. However, this claim is incorrect. Notably, Lallensack et al. (2022), which is cited in the author’s paper, is actually the first application of machine-learning techniques at the academic level for large ornithopod dinosaur footprints. Lallensack et al’s focus was on using machine learning to distinguish between tridactyl tracks formed by theropods and ornithopods (not sauropods as stated on page 15). To address this discrepancy, I have two recommendations: 1) Introduce the Lallensack et al. study in the revised manuscript’s introduction to highlight the novelty and significance of the method employed. Moreover, clarify the distinctions between Lallensack et al.’s work and the current study, as Lallensack et al. specifically addressed the problem of misidentifying ornithopod tracks as theropodan and vice versa. 2) State that the present study represents the first application of machine-learning techniques at the academic level for classifying large ornithopod dinosaur footprints. It is important to clarify that this paper does not provide a comprehensive evaluation (there are large-bodied ornithopod tracks that were not included in Díaz-Martínez et al study) but rather follows the established work of Díaz-Martínez et al. (2015), which is widely accepted in the field.*

- Agreed. We have changed our “first” statement limiting to verification of the ichnotaxonic assignments of large ornithopod dinosaur footprints. Then, the differences between Lallensack et al (2022) and our study was introduced to the Results and Discussion section as “In addition, employing machine learning in fossil studies can minimize subjectivity and reduce information loss when delineating fossil footprints. Lallensack et al. [58] were the first to introduce a machine learning technique to differentiate between tridactyl tracks formed by theropods and ornithopods. They trained a deep convolutional neural network (DCNN) using fossil silhouettes. Since our study emphasizes the classification of ornithopod ichnotaxa as established by Díaz-Martínez et al. [3], it's crucial for researchers to meticulously choose the ornithopod footprints before deploying the model. However, both studies are inherently limited by the information available from interpretive fossil outlines. This constraint can be addressed by broadening the database to encompass three-dimensional fossil morphology [58].”

*Other comments/suggests accompany pdf*

- We closely followed your comments given in the annotated PDF when revising the manuscript.

*Reviewer #2:*

*This is an interesting study of an emerging approach to comparing shapes of tridactyl footprints. While I have some reservations about how well outlines of footprints capture their overall shape, I nonetheless think your approach is worth pursuing.*

- Thank you for taking the time to review our study and for recognizing the potential of our approach. We understand and share your concerns about the limitations of footprint outlines in capturing their overall shape. However, we believe that with further refinement and additional data sources, this method can offer valuable insights. Your feedback is invaluable, and we will certainly consider it as we continue our research. Once again, thank you for your constructive comments.
